# Study protocol for a randomized trial comparing two electroacupuncture waveforms for different severity groups of Bell palsy

**DOI:** 10.3389/fneur.2024.1471605

**Published:** 2024-11-20

**Authors:** Zhiyuan Bian, Jiawei Wang, Fei Fang, Binyan Yu, Yan Shi, Yijia Wan, Mei Hong, Conghua Ji, Xiaomei Shao, Yi Liang, Jianqiao Fang, Jing Sun

**Affiliations:** ^1^The Third Clinical College of Zhejiang Chinese Medical University, Hangzhou, China; ^2^Department of Acupuncture and Moxibustion, The First Affiliated Hospital of Zhejiang Chinese Medical University, Hangzhou, China; ^3^Department of Acupuncture and Massage, Hangzhou First People’s Hospital, Hangzhou, China; ^4^School of Public Health, Zhejiang Chinese Medical University, Hangzhou, China

**Keywords:** Bell palsy, electroacupuncture, waveform, randomized, pilot trial, protocol

## Abstract

**Background:**

Bell palsy (BP) is the most common cause of acute peripheral facial palsy which leads to functional and esthetic disturbances in patients and has a negative influence on daily living. Electroacupuncture (EA) has been considered an alternative treatment for improving facial function in patients with BP. However, there is no agreement on the preferred waveform type of the EA for treating BP.

**Methods:**

This is a study protocol for a pilot randomized, two-arm, three-center, clinical trial at the Third Affiliated hospital of Zhejiang Chinese Medical University, the First Affiliated hospital of Zhejiang Chinese Medical University, and the Hangzhou First People’s Hospital. The trial began in November 2023 and is expected to end in December 2025. Sixty patients with BP whose electroneurography (ENoG) value is at least 20% and 60 patients with BP whose ENoG value is less than 20% will be randomly assigned to the low frequency continuous waveform group or the intermittent waveform group in a 1:1 ratio. Participants will receive 4 weeks of EA treatment and clinical assessments. The primary outcome is the change from baseline score of the Facial Nerve Grading System 2.0. The secondary outcomes include the change from baseline score of the Sunnybrook grading scale and the change from baseline amplitude of the compound muscle action potential of the affected side in the ENoG tests.

**Discussion:**

This is the first study protocol to compare the treatment effect and safety of EA with low frequency continuous waveform and intermittent waveform for different severity groups of BP. This study will contribute to subsequent studies for exploring optimal EA parameters for BP treatment.

**Clinical trial registration:**

ClinicalTrials.gov, NCT06063954.

## 1 Introduction

Bell palsy (BP), also known as idiopathic facial paralysis, is a neurological condition that affects facial nerve function with unclear pathogenesis (1). BP is the most common cause of acute facial palsy, accounting for approximately 75% of all cases (2, 3). The reported annual incidence of BP ranges from 11.5 to 53.3 per 100,000 persons in different populations (4, 5). Functional and esthetic impairments are common among individuals with BP, as the most prominent symptom of the condition is the sudden weakness of facial muscles, which usually reaches peak severity within 72 h, leading to a drooping eyelid and corner of the mouth and even the complete loss of facial movement (6). And other symptoms may include decreased taste, hyperacusis, and pain around the ear or jaw (7, 8). In addition, patients with BP may face a heightened risk of psychological disorders, including anxiety and depression, and may also experience a decrease in overall quality of life (9).

Bell palsy has a generally fair prognosis, with most patients experiencing important improvement within 3 weeks (10). Even without intervention, approximately 70% of patients achieve complete recovery within 6 months (2). However, permanent paresis or paralysis occurs in a minority of the patients. The use of electroneurography (ENoG) to objectively measure the severity of facial nerve damage can aid in predicting prognosis (11–13). In ENoG testing, surface electrodes record the compound muscle action potential (CMAP) of facial muscles following electrical stimulation to the main trunk of the facial nerve. The ENoG value is calculated as the ratio of the CMAP amplitude on the impaired side to that on the normal side (14). A previous study showed that an ENoG value of less than 20% predicted a recovery period of at least 4 months, with the possibility of incomplete recovery in the worst cases (15).

Among multiple treatment options for BP, corticosteroids are typically used in the acute phase because of their potent anti-inflammatory effect, with strong evidence demonstrating the benefit for adult patients (16). However, effective treatments for BP in the non-acute stage remain unclear (17). In China, acupuncture has been used in a variety of neurological conditions, including BP. According to a rigorous randomized control trial, manipulating needles to strengthen stimulation during acupuncture provided a stronger therapeutic effect than acupuncture without manipulation in treating BP (18). Electroacupuncture (EA), a treatment that combines traditional acupuncture and electrical stimulation, offers the benefit and convenience of enhancing stimulation through consistent delivery of electrical pulses through the needles. A systematic review showed that EA is more effective than manual acupuncture for BP (19). In clinical practice, controversy exists over whether EA is appropriate for use during the acute stage of BP. Although limited evidence has shown that EA initiation from the acute stage of BP may shorten the recovery time, there are concerns that excessive physical stimulation during the period of nerve degeneration may worsen nerve damage (20, 21). In contrast, the safety of EA when used in the non-acute phase is widely recognized, and a large number of studies have reported its positive effects at this stage (22–24). Notably, as a crucial parameter in EA treatment, the electrical stimulation waveform varies across studies with no consensus on the most effective option. These waveforms mainly include the low frequency or high frequency continuous wave (25), which delivers electrical pulses consistently with a certain frequency, the sparse-dense wave (26), which generates alternating low frequency and high frequency electrical stimulation, and the intermittent wave (23), which delivers electrical pulses in a repeated on–off manner. The lack of a standardized protocol for EA when treating BP poses an obstacle to practitioners.

Previous studies have shown that EA with different waveforms have varying treatment effects. The low frequency continuous waveform at 2 Hz was found to be more beneficial for nerve regeneration than the high frequency continuous waveform at 100 Hz and the sparse-dense waveform at 2/100 Hz (27–29). EA stimulation with the intermittent waveform was found to increase muscle size and enhance muscle excitability (30, 31), and 30–50 Hz are a common frequency range for intermittent waveform (32). While both the low frequency continuous waveform and the intermittent waveform of EA have been proven to have positive effects on neuromuscular function, it is yet to be determined which waveform is more beneficial for the treatment of BP. In addition, the prognosis of BP is significantly impacted by the level of facial nerve damage, making it valuable to investigate the treatment effects on different severity groups (13). Therefore, we designed this randomized trial to compare the treatment effect and safety of these two waveforms of EA stimulation for individuals with moderate or severe BP, categorized by their ENoG value of at least 20% or below 20% (15). This pilot study will assess the feasibility and safety of the treatment protocol, and has the potential to provide preliminary basis for subsequent research.

## 2 Study objectives

This study is a protocol for a pilot randomized trial aimed at comparing the treatment effect and safety of EA with two different waveforms, the low frequency continuous waveform and the intermittent waveform, for different severity groups of BP.

## 3 Materials and methods

The study protocol follows the Standard Protocol Items: Recommendations for Interventional Trials statement (33), and the trial will be in accordance with the Declaration of Helsinki and follow the Consolidated Standards of Reporting Trials (34) and the Revised Standards for Reporting Interventions in Clinical Trials of Acupuncture (35) guidelines. The trial was registered in ClinicalTrials.gov (ID: NCT06063954).

### 3.1 Trial design and setting

**Figure 1:**
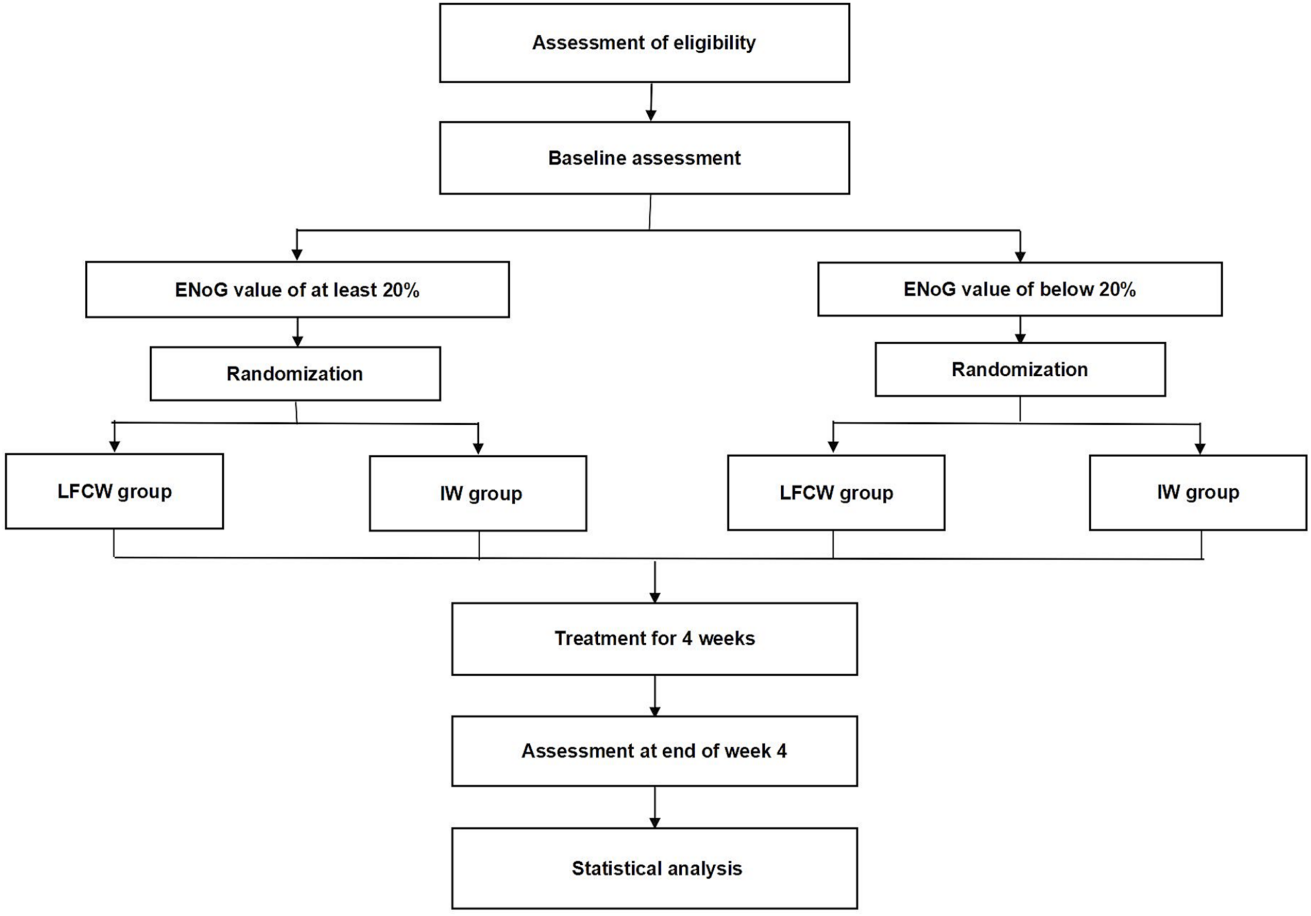
Trial procedure. LFCW, Low frequency continuous waveform; IW, Intermittent waveform.

**Figure 2:**
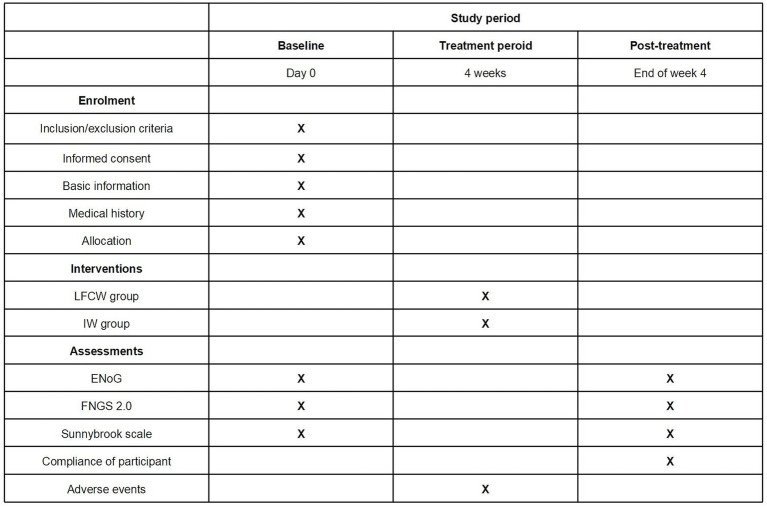
Schedule of allocation, treatment, and measurement. LFCW, Low frequency continuous waveform; IW, Intermittent waveform.

This trial will be conducted at the Third Affiliated Hospital of Zhejiang Chinese Medical University, the First Affiliated Hospital of Zhejiang Chinese Medical University, and Hangzhou First People’s Hospital. The trial was approved and regularly reviewed by the research ethics committees of all three hospitals. The trial began in November 2023 and is expected to end in December 2025. Recruitment strategies included print posters displayed in outpatient departments and online posters on the WeChat social media platform. The intervention included a 4-week EA session, with different waveforms in each group. The trial procedure is shown in Figure 1, and schedule for allocation, treatment, and measurement is shown in Figure 2.

### 3.2 Participants

Potential eligible participants presenting with facial palsy will be recruited from the outpatient units of the acupuncture and moxibustion departments. Each eligible participant will receive a comprehensive explanation of the trial protocol before signing the informed consent. Eligible and consenting participants at each severity level will be randomly allocated to receive a 4-week EA treatment with the low frequency continuous wave pattern or the intermittent wave pattern in a 1:1 ratio.

Participants meeting the following criteria will be included: (1) Diagnosed as BP by neurology clinician; (2) Facial Nerve Grading System 2.0 (FNGS 2.0) is at least 15 on day 21 after the onset of BP; (3) Age between 18 and 65 years old; (4) Received prednisolone within 72 h after the initial symptoms of BP, the dose used was 60 mg per day for 5 days and then tapered by 10 mg per day; and (5) Signed informed consent.

Participants with any of the following conditions will be excluded: (1) Facial palsy caused by other diseases; (2) Diagnosed as Ramsey-Hunt syndrome; (3) Presenting bilateral facial palsy; (4) History of previous facial palsy; (5) Presenting with facial spasm, facial synkinesis, or contracture on day 21 after the onset of BP; (6) History of surgery on face; (7) Combined with uncontrolled diabetes mellitus, uncontrolled hypertension, serious conditions involving the heart, liver, or kidney, cognitive impairment, aphasia, or mental disorders; (8) Wearing a pacemaker; and (9) Pregnant or lactating patients.

The basic information and clinical data of each participants will be recorded using a case report form. And at each research center, an independent researcher will enter the data into an electronic data capture system (DAP Software Co., Ltd., China) in a timely manner.

### 3.3 Randomization and allocation concealment

In total, 120 participants are planned to be enrolled from three clinical centers. Based on the patient volume and available resources at each center, we plan to recruit 70 participants from the First Affiliated Hospital of Zhejiang Chinese Medical University, 30 participants from the Third Affiliated Hospital of Zhejiang Chinese Medical University, and 20 participants from Hangzhou First People’s Hospital. For both severity levels, participants in all three clinical centers will be randomly assigned in a 1:1 ratio to the low frequency continuous wave group or the intermittent wave group using the same central computer system (DAP Software Co., Ltd., China) by an independent researcher at each clinical center. Allocation to treatment groups will be under the control of this system automatically, and therefore, will not be influenced by either researchers or participants.

### 3.4 Blinding

In each clinical center, EA treatment will be provided by two acupuncturists, one for needle insertion and manipulation and the other for operation of the EA device. Acupuncturists who operate EA devices will not be blinded to the treatment allocation because the two treatment groups will receive EA treatment with different waveforms, and the EA device can only be operated manually. Acupuncturists who perform needle insertion and manipulation, participants, outcome assessors, and researchers for data management will be blinded to treatment assignment.

### 3.5 Intervention

**Figure 3:**
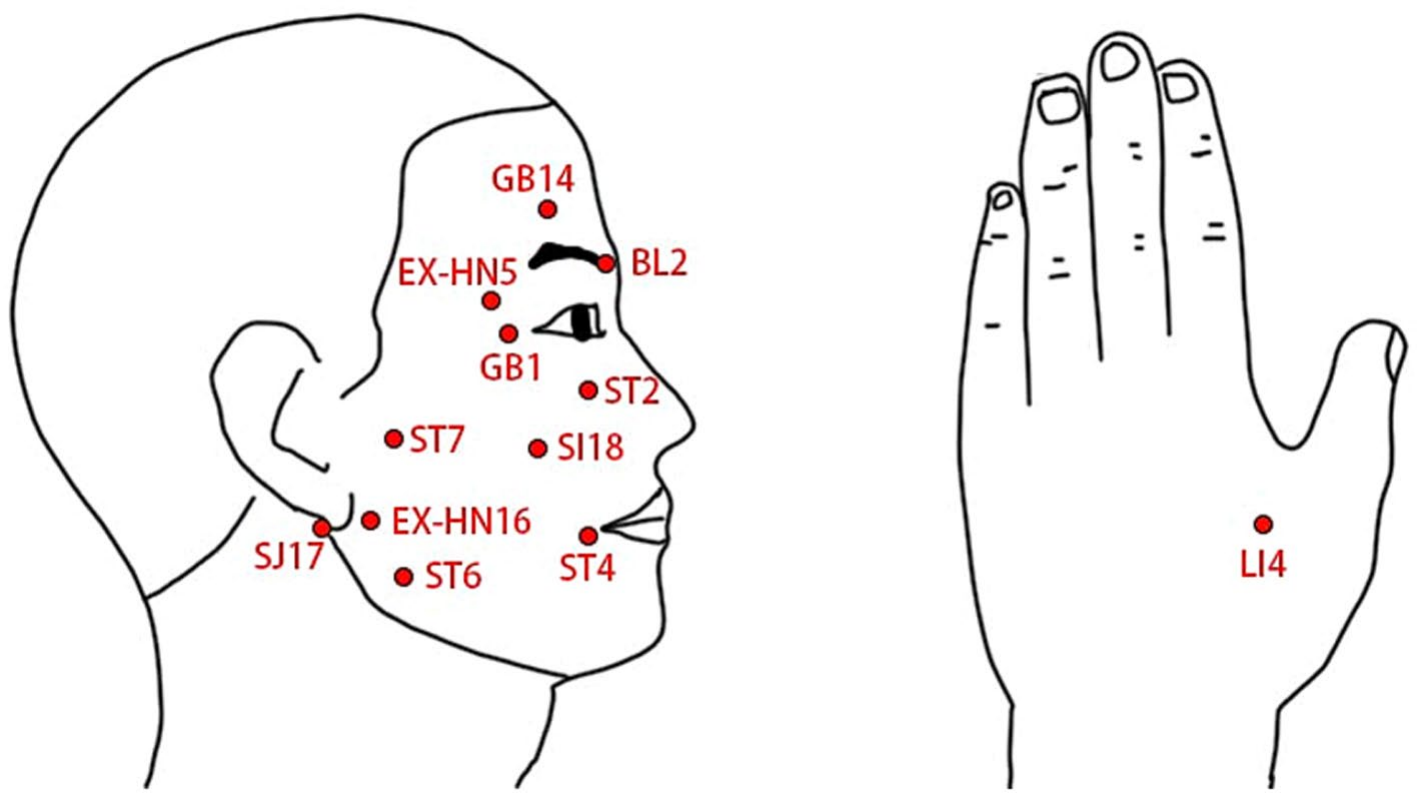
Illustration of the locations of selected acupoints.

The two treatment groups will use the same acupoint prescription and needling method. Based on both traditional Chinese medicine literature and clinical experience, acupoint prescription will adhere to the principle of choosing both local and distal acupoints: BL2, GB1, GB14, ST2, ST4, ST6, ST7, SI18, SJ17, EX-HN5, EX-HN16, on the affected side, and LI4, bilaterally, as shown in Table 1 and Figure 3. Participants will be in a supine position, and after the skin sterilization, sterile and disposable acupuncture needles with 0.25 mm in diameter and 40 mm in length (Hwato Brand, Suzhou Medical Instrument, China) will be inserted into the prescript acupoints at a depth of 5 mm–30 mm and manually manipulated to elicit de-qi sensation. After needle insertion and manipulation, an SDZ-IIB EA device (Hwato Brand, Suzhou Medical Instrument, China) will be used to generate electrical stimulation, with a pair of electrodes connected to BL2 and GB1, and the other pair of electrodes connected to ST4 and ST6. In the low frequency continuous waveform group, EA with the 2 Hz continuous waveform will be used for 20 min, and in the Intermittent waveform group, EA with the 40 Hz intermittent waveform will be used for 20 min, the choose of 40 Hz for intermittent waveform stimulation was based on clinical experience and previous study (32). After the EA stimulation is completed, all needles will be removed to end the single treatment session. Besides EA treatment, no other treatment will be used for participants during the treatment period. Based on previous study on acupuncture therapy for BP (36), we set the treatment period to 4 weeks, during which participants will receive treatment three times a week, totaling 12 sessions.

**Table 1:** Locations of acupuncture points.

Acupoint	Location
BL2 Cuanzhu	In the depression at the medial end of the eyebrow.
GB1 Tongziliao	In the depression at 0.5 cun lateral to the outer canthus of the eye.
GB14 Yangbai	At 1 cun superior to the eyebrow, directly superior to the center of the pupil.
ST2 Sibai	In the infraorbital foramen.
ST4 Dicang	At 0.4 cun lateral to the angle of the mouth.
ST6 Jiache	At one fingerbreadth anterosuperior to the angle of the mandible.
ST7 Xiaguan	In the depression between the midpoint of the inferior border of the zygomatic arch and the mandibular notch.
SI18 Quanliao	At inferior border of the zygomatic bone, in the depression directly inferior to the outer canthus of the eye.
SJ17 Yifeng	In the depression anterior to the inferior end of the mastoid process.
EX-HN5 Taiyang	In the depression at one fingerbreadth posterior to the midpoint between the lateral end of the eyebrow and the outer canthus.
EX-HN16 Qianzheng	At 0.5–1 cun anterior to the auricular lobe.
LI4 Hegu	On the radial side of the midpoint of the second metacarpal bone.

### 3.6 Outcome measurement

#### 3.6.1 Primary outcome

*Change from baseline score of the Facial Nerve Grading System 2.0 at week 4.* The Facial Nerve Grading System 2.0 (FNGS 2.0) is an update version of the original House-Brackmann (H-B) scale (37). A study have shown that the inter-rater consistency when using the H-B scale is significantly influenced by the experience level of the assessors. In contrast, the FNGS 2.0 demonstrated better repeatability and consistency, independent of the assessor’s experience, indicating improved accuracy (38). Therefore, this pilot trial, we will use the FNGS 2.0 instead of the original H-B scale. In the FNGS 2.0, function of four facial regions including brow, eye, nasolabial fold, and oral commissure, and the global impression of secondary movement are separately assessed. Therefore, confusion can be addressed when different regions do not conform to a certain grade on the original H-B scale. Each area is assigned a score of 1–6, and the severity of secondary movement is assigned a score of 0–3, with higher score indicating poorer function. The final score is determined by combining the four regional and secondary movement scores. The primary outcome of this trial will be the change from baseline score of FNGS 2.0 at week 4.

#### 3.6.2 Secondary outcome

*Change from baseline score of the Sunnybrook grading scale at week 4.* The Sunnybrook grading scale includes three subscales: the resting symmetry subscale, the symmetry of voluntary movement subscale, and the synkinesis subscale (39). Poorer function is indicated by a higher score in the resting symmetry subscale and the synkinesis subscale, and a lower score in the symmetry of voluntary movement subscale. The final score is calculated by subtracting the total resting symmetry score and total synkinesis score from the total voluntary movement score. In this trial, the change from baseline score of each subscale and the composite score will be calculated at week 4.

*Change from baseline value of the amplitude the CMAP of the affected side at week 4.* In the ENoG test, CMAP is the summated action potentials of the stimulated motor endplates following electrical stimulation to the facial nerve. In this trial, the ENoG test will be performed, and the change from baseline value of the amplitude of the CMAP of the affected side will be calculated at week 4 (40).

### 3.7 Compliance assessment and safety assessment

Participants’ compliance will be assessed at the end of week 4. Participants with scores of less than 80 will be considered to have poor compliance.


$$\mathrm{Compliance}\;\mathrm{score}=\frac{\mathrm{Number}\;\mathrm{of}\;\mathrm{actual}\;\mathrm{treatment}\;\mathrm{sessions}}{\mathrm{Number}\;\mathrm{of}\;\mathrm{required}\;\mathrm{treatment}\;\mathrm{sessions}}\times 100\%$$

Participants will be instructed to report adverse events during the study period. Common adverse events associated with acupuncture include dizziness, paresthesia, needle-site bleeding and pain, etc. All adverse events will be recorded and assessed by the researchers in detail, including symptoms, occurrence time, duration, severity, management, resolution, and causality relationship with the intervention (41, 42).

### 3.8 Sample size

This trial will compare the two EA waveforms in different BP severity groups. However, studies with comparable designs are lacking. According to a study by Sim and Lewis (43, 44), a pilot trial of at least 55 participants would minimize the overall sample size for small to medium standardized effect sizes. Therefore, we will recruit 120 participants in total, with 60 participants at each severity level. And participants will be assigned to the two treatment groups in a 1:1 ratio.

### 3.9 Statistical analysis

Statistical analysis will be performed using SAS software version 9.3 (SAS Institute Inc., United States). Numerical data with normal distribution will be presented as mean ± standard deviation, whereas non-normally distributed data will be shown as median and interquartile range. For categorical data, the frequency and percentage of each category will be presented. The Student’s *t* test or Mann–Whitney U test will be used for comparisons of numerical variables, and the chi-squared test or Fisher’s exact test will be used for comparisons of categorical variables. All statistical tests will be two-sided, with the significance level set at 0.05. Both intention-to-treat and per-protocol populations will be analyzed. Missing data will be substituted using the last observation carried forward method. After the primary statistical analysis, a power analysis will be conducted using G*Power software version 3.1.9.7 (Heinrich Heine University Düsseldorf, Germany) to evaluate the statistical power of the study given the sample sizes and the obtained effect sizes.

## 4 Discussion

In the treatment of BP, administering corticosteroids during the acute phase is crucial to decrease inflammation and edema of the facial nerve. After the acute phase, the treatment focus shifts to promoting nerve repair and restoring facial muscle function (32, 45). Through animal studies, a number of possible mechanisms of EA in promoting facial nerve recovery have been identified. In rabbit models of facial nerve injury, EA was found to increase a variety of neurotrophic factors, including neurotrophin-3 and ciliary neurotrophic factor, which accelerate axon outgrowth in damaged nerves (46, 47). In addition, EA was found to activate neuronal cell adhesion molecules, including E-cadherin and calmodulin, which are involved in facilitating adhesion, differentiation, and guidance during neuronal growth (48). The use of EA in the management of BP has been prevalent in China. Although published systematic reviews and a large number of clinical trials have reported its positive effect on BP, high-quality evidence is lacking (19, 24). The absence of a standardized protocol for EA treatment hindered both the clinical use and evaluation of the efficacy and safety of EA in the treatment of BP.

There are many parameters that influence the effects of EA, including intensity, duration, waveform, and frequency. Our study focuses on the waveform, which encompasses both frequency and the distinction between continuous and intermittent stimulation. Since waveform is a primary parameter to consider when operating EA devices, comparing the effects of different waveforms carries significant clinical implications. Notably, when using different waveforms in clinical settings, the chosen electrical stimulation frequencies often vary. For instance, continuous waves below 30 Hz are typically classified as low frequency, while those at 30 Hz and above are classified as high frequency (49). Although there are variations in frequency selection across studies, 2 Hz has been widely researched in the low frequency range, which havs been found to aid nerve regeneration (27). Therefore, we selected 2 Hz for the low frequency continuous wave group. For the intermittent waveform, based on clinical experience and previous literature (32), frequencies between 30 Hz and 50 Hz are commonly chosen, leading us to select 40 Hz for the intermittent group in this pilot study. In future research, it will be meaningful to continue investigating the effects of different frequency electrical stimulation under the same waveform conditions. For example, 20 Hz is also a commonly used frequency in experimental studies (50, 51). These explorations will help identify the optimal EA parameters in the treatment of BP.

The recovery time of the facial nerve function varies among patients with BP. Patients with quick recovery can restore complete function within 2–3 weeks since the onset of BP, while in severe cases, patients took 6 month to achieve full recovery or were left with varying degrees of sequelae (2). Prolonged facial asymmetry symptoms was reported to have more severe impact on daily living (52). Therefore, in this pilot trial, we plan to focus on patients with BP who do not experience a quick recovery. We will recruit patients with a score of at least 15 on FNGS 2.0 at day 21 after the onset of BP, with scores of 15–24 on FNGS 2.0 corresponding to Grade IV (moderately severe dysfunction) to VI (total paralysis) on the original H-B scale. We plan to investigate the efficacy and safety of the two waveforms of EA in two severity groups categorized by an ENoG value of at least 20% or below 20%, the results of this study have the potential to guide the clinical use of EA for different degrees of severity of BP.

Our study has several limitations. First, promoting the quick recovery of facial nerve function is important for all patients with BP, however, we will only include participants who presented with moderately severe to complete paralysis at day 21 after the onset of BP. Thus, the effects of EA in cases of mild severity will not be evaluated in this study. Second, in this study, we will not include a conventional acupuncture group or sham EA group. We expect to obtain the better EA waveform for treating BP in this trial, and we plan to verify its efficacy and safety by comparing it with conventional acupuncture or sham EA in future studies. Third, though in previous study of acupuncture therapy for BP, differences of symptom improvement among different treatment groups were observed after 4-week treatment (36), however, for patients with severe facial nerve impairment, the 4-week treatment period may be relative short. Moreover, we did not establish a post-treatment follow-up period to assess the long-term effects of the treatment. In future research, it is worth to explore the effect of longer treatment period, and also the long-term effect of EA for BP. Finally, although we plan to recruit patients from three hospitals to screen more potential participants, all of these hospitals are in the same city, which may lead to poor representativeness of the participants.

In conclusion, we plan to conduct a two-arm, three-center pilot trial to compare the efficacy and safety of two EA waveforms for the treatment of BP. The findings of this study will contribute to future studies for exploring optimal EA parameters for BP.
